# DprA Is Essential for Natural Competence in *Riemerella anatipestifer* and Has a Conserved Evolutionary Mechanism

**DOI:** 10.3389/fgene.2019.00429

**Published:** 2019-05-17

**Authors:** Li Huang, Xiu Tian, Mafeng Liu, Mingshu Wang, Francis Biville, Anchun Cheng, Dekang Zhu, Renyong Jia, Shun Chen, Xinxin Zhao, Qiao Yang, Ying Wu, Shaqiu Zhang, Juan Huang, Bin Tian, Yanling Yu, Yunya Liu, Ling Zhang, Leichang Pan, Mujeeb Ur Rehman, Xiaoyue Chen

**Affiliations:** ^1^Institute of Preventive Veterinary Medicine, Sichuan Agricultural University, Chengdu, China; ^2^Research Center of Avian Disease, College of Veterinary Medicine, Sichuan Agricultural University, Chengdu, China; ^3^Key Laboratory of Animal Disease and Human Health of Sichuan Province, College of Veterinary Medicine, Sichuan Agricultural University, Chengdu, China; ^4^Institut Pasteur, Paris, France

**Keywords:** *Riemerella anatipestifer*, *dprA*, natural competence, EMSA, evolution

## Abstract

*Riemerella anatipestifer* ATCC11845 (RA ATCC11845) is naturally competent. However, the genes involved in natural transformation in this species remain largely unknown. Bioinformatic analysis predicts that DprA of RA (DprA_Ra_) has three domains: a sterile alpha motif (SAM), a Rossmann fold (RF) domain and a Z-DNA-binding domain (Zα). Inactivation of *dprA* abrogated natural transformation in RA ATCC11845, and this effect was restored by the expression of *dprA in trans*. The *dprA* with SAM and RF domains of *Streptococcus pneumoniae* and the *dprA* with RF and Zα domains of *Helicobacter pylori* was able to restore natural transformation in the RA ATCC11845 *dprA* mutant. An Arg123 mutation in the RF domain of *R. anatipestifer* was not able to restore natural transformation of the RA ATCC11845 *dprA* mutant. Furthermore, DprA^R123E^ abolished its ability to bind DNA, suggesting that the RF domain is essential for the function of DprA. Finally, the *dprA* of *Fusobacterium naviforme* which has not been reported to be natural competent currently was partially able to restore natural transformation in RA ATCC11845 *dprA* mutant. These results collectively suggest that DprA has a conserved evolutionary mechanism.

## Introduction

Natural transformation involves the acquisition of naked DNA by a bacterium from the extracellular environment and integrated DNA into their genome, and genetic competence is the ability to undergo natural transformation (Chen and Dubnau, 2004). At present, at least 82 bacterial species have been shown to be naturally transformable (Johnston et al., 2014; Mell and Redfield, 2014). The process of natural transformation involves two steps: DNA uptake and DNA processing (Johnston et al., 2014). Gram-positive and Gram-negative bacteria rely on highly similar DNA-uptake systems (Johnston et al., 2014). Transformable Gram-negative species use proteins that are related to those involved in the assembly of type II secretion systems and type IV pili (T4P) (Hobbs and Mattick, 1993; Dubnau, 1999). It has been proposed that structure similar to T4P known as a competence pseudopilus participates in DNA transport in Gram-positive bacteria (Dubnau, 1999). Once dsDNA has entered the periplasm, one strand of the dsDNA is degraded, and the other strand is internalized via a ComEC transmembrane channel (Draskovic and Dubnau, 2005). After internalization, the transforming ssDNA is bound to the transformation-dedicated recombination mediator protein (RMP) DprA, which loads the homologous recombinase RecA to promote homologous recombination (Mortier-Barriere et al., 2007; Yadav et al., 2013).

*Riemerella anatipestifer* (*R. anatipestifer*, RA) is a Gram-negative bacterium that causes septicemic diseases in ducks, geese, turkeys, and other birds (Huang et al., 2017). At present, at least 21 serotypes of *R. anatipestifer* have been identified (reference). The extensive use of antibiotics for the treatment and prevention of serositis and septicemia has resulted in multidrug resistance in *R. anatipestifer* (Zhong et al., 2009; Luo et al., 2015, 2018; Huang et al., 2017; Zhang et al., 2017; Zhu et al., 2018), and at least 33 relevant bacterial genomes with genome sizes ranging from 2.09 to 2.43 Mb have been sequenced from different isolates (Wang X. et al., 2014; Song et al., 2016; Zhu et al., 2016). Sequence analysis of RA ATCC11845, RA CH-1, and RA CH-2 showed that RA CH-1 is 140,000 bp larger than the two other strains and there are so many resistance genes in this region (Wang X. et al., 2014). However, the reason for this genome diversity and the mechanisms underlying multidrug resistance remain largely unknown. In our previous study, we showed that *R. anatipestifer* is naturally competent (Liu et al., 2017). At present, the machinery involved in the taking up of exogenous dsDNA involves T4P or T4SS (Johnston et al., 2014). However, no homologs of T4P and T4SS were found in the genome of *R. anatipestifer* through sequence comparison. Several genes which was annotated as *recA*, *dprA*, *comM*, and *comEC*, respectively, in the genome of *R. anatipestifer* are predicted to be involved in transporting DNA across inner membrane and homologous recombination (Liu et al., 2017). Bioinformatic analysis showed that the gene *RA0C_1073* of RA ATCC11845 encodes a putative DprA with low identity to the DprA found in other bacteria. In addition, RA ATCC11845 is the first bacteria in *Flavobacteriaceae* that has been reported to have natural competence. In this study, we sought to provide a functional characterization of DprA and its mechanism in the natural transformation of RA ATCC11845. This information will be helpful for determining the mechanism of natural transformation in bacteria occurring in *R. anatipestifer* and other members of *Flavobacteriaceae*.

## Materials and Methods

### Bacterial Strains, Plasmids and Primers

The bacterial strains and plasmids used in this study are shown in Table 1. The primers are shown in Table 2.

**Table 1:** Strains and plasmids used in this study.

*Riemerella anatipestifer* strains	Genotype or description	Source or references
RA ATCC11845	RA ATCC11845, Km^R^	Laboratory collection
RA ATCC11845Δ*dprA*::*Erm*	RA ATCC11845Δ*dprA*, Erm^R^	This study
RA ATCC11845 (pLMF03::*dprA*)	RA ATCC11845 carrying pLMF03::*dprA*, Amp^R^ Cfx^R^	This study
RA ATCC11845 (pLMF03::*dprA*^R123E^)	RA ATCC11845 carrying pLMF03::*dprA*^R123E^, Amp^R^ Cfx^R^	This study
RA ATCC11845 (pLMF03::*dprA*-SAM-RF)	RA ATCC11845 carrying pLMF03::*dprA*-SAM-RF, Amp^R^ Cfx^R^	This study
RA ATCC11845 (pLMF03::*dprA*-RF-Zα)	RA ATCC11845 carrying pLMF03::*dprA*-RF-Zα, Amp^R^ Cfx^R^	This study
RA ATCC11845 (pLMF03::Sp-*dprA*)	RA ATCC11845 carrying pLMF03::Sp-*dprA*, Amp^R^ Cfx^R^	This study
RA ATCC11845 (pLMF03::Hp-*dprA*)	RA ATCC11845 carrying pLMF03::Hp-*dprA*, Amp^R^ Cfx^R^	This study
RA ATCC11845 (pLMF03::Fn-*dprA*)	RA ATCC11845 carrying pLMF03::Fn-*dprA*, Amp^R^ Cfx^R^	This study
RA ATCC11845Δ*dprA*::*Erm* (pLMF03::*dprA*)	RA ATCC11845Δ*dprA* carrying pLMF03::*dprA*, Erm^R^ Amp^R^ Cfx^R^	This study
RA ATCC11845Δ*dprA*::*Erm* (pLMF03::*dprA*^R123E^)	RA ATCC11845Δ*dprA* carrying pLMF03::*dprA*^R123E^, Erm^R^ Amp^R^ Cfx^R^	This study
RA ATCC11845Δ*dprA*::*Erm* (pLMF03::*dprA*-SAM-RF)	RA ATCC11845Δ*dprA* carrying pLMF03::*dprA*-SAM-RF, Erm^R^ Amp^R^ Cfx^R^	This study
RA ATCC11845Δ*dprA*::*Erm* (pLMF03::*dprA*-RF-Zα)	RA ATCC11845Δ*dprA* carrying pLMF03::*dprA*-RF-Zα, Erm^R^ Amp^R^ Cfx^R^	This study
RA ATCC11845Δ*dprA*::*Erm* (pLMF03::Sp-*dprA*)	RA ATCC11845Δ*dprA* carrying pLMF03::Sp-*dprA*, Erm^R^ Amp^R^ Cfx^R^	This study
RA ATCC11845Δ*dprA*::*Erm* (pLMF03::Hp-*dprA*)	RA ATCC11845Δ*dprA* carrying pLMF03::Hp-*dprA*, Erm^R^ Amp^R^ Cfx^R^	This study
RA ATCC11845Δ*dprA*::*Erm* (pLMF03::Fn-*dprA*)	RA ATCC11845Δ*dprA* carrying pLMF03::Fn-*dprA*, Erm^R^ Amp^R^ Cfx^R^	This study

***Escherichia coli* strains**	**Genotype or description**	**Source or references**

DH5α	*E. coli* DH5α, cloning host cell	Laboratory collection
XL1-blue	*E. coli* XL1-blue, cloning host cell	Laboratory collection
Rosetta	*E. coli* Rosetta, expressing host cell	Laboratory collection
Rosetta (pET30a)	*E. coli* Rosetta carrying pET30a, Km^R^	This study
Rosetta (pET30a::*dprA*-s)	*E. coli* Rosetta carrying pET30a::*dprA*, Km^R^	This study
Rosetta (pET30a::*dprA*^R123E^-s)	*E. coli* Rosetta carrying pET30a::*dprA*^R123E^-s, Km^R^	This study
S17-1	*Thi-1 thr leu tonA lac Y supE recA*::RP4-2-Tc::Mu Km^R^	Miller and Mekalanos, 1988
S17-1 (pLMF03::*dprA*)	S17-1 carrying pLMF03::*dprA*, Amp^R^ Cfx^R^	This study
S17-1 (pLMF03::*dprA*^R123E^)	S17-1 carrying pLMF03::*dprA*^R123E^, Amp^R^ Cfx^R^	This study
S17-1 (pLMF03::*dprA*-SAM-RF)	S17-1 carrying pLMF03::*dprA*-SAM-RF, Amp^R^ Cfx^R^	This study
S17-1 (pLMF03::*dprA*-RF-Zα)	S17-1 carrying pLMF03::*dprA*-RF-Zα, Amp^R^ Cfx^R^	This study
S17-1 (pLMF03::Sp-*dprA*)	S17-1 carrying pLMF03::Sp-*dprA*, Amp^R^ Cfx^R^	This study
S17-1 (pLMF03::Hp-*dprA*)	S17-1 carrying pLMF03::Hp-*dprA*, Amp^R^ Cfx^R^	This study
S17-1 (pLMF03::Fn-*dprA*)	S17-1 carrying pLMF03::Fn-*dprA*, Amp^R^ Cfx^R^	This study

**Plasmids**	**Genotype or description**	**Source or references**

pET30a	pBR322 lacZ, IPTG-inducible promoter, Km^R^	Laboratory collection
pET30a::*dprA*-s	pET30a carrying *dprA* adding his tag from RA ATCC11845, Km^R^	This study
pET30a::*dprA*^R123E^-s	pET30a carrying Arg123 site-directed mutant *dprA* adding his tag of RA ATCC11845, Km^R^	This study
pLMF03	*B739_0921* promoter, oriColE1, ori pRA0726, Amp^R^ Cfx^R^	Liu et al., 2016
pLMF03::*dprA*	pLMF03 carrying *dprA* from RA ATCC11845, Amp^R^ Cfx^R^	This study
pLMF03::*dprA*^R123E^	pLMF03 carrying Arg123 site-directed mutant *dprA* of RA ATCC11845, Amp^R^ Cfx^R^	This study
pLMF03::Sp*-dprA*	pLMF03 carrying *dprA* from *S. pneumoniae*, Amp^R^ Cfx^R^	This study
pLMF03::Hp*-dprA*	pLMF03 carrying *dprA* from *H. pylori*, Amp^R^ Cfx^R^	This study
pLMF03::Fn*-dprA*	pLMF03 carrying *dprA* from *F. naviforme*, Amp^R^ Cfx^R^	This study
pLMF03::*dprA*-SAM-RF	pLMF03 carrying SAM and RF domains of *dprA* from RA ATCC11845, Amp^R^ Cfx^R^	This study
pLMF03::*dprA*-RF-Zα	pLMF03 carrying RF and Zα domains of *dprA* from RA ATCC11845, Amp^R^ Cfx^R^	This study

*Amp^R^, ampicillin resistance; Km^R^, kanamycin resistance; Cfx^R^, cefoxitin resistance.*

**Table 2:** Primers used in this study.

Primers	Sequence	Organism
16SrRNAP1	CGAAAGTGATAAGTTAGCCACCT	RA ATCC11845
16SrRNAP2	GCAGCACCTTGAAAATTGTCC	RA ATCC11845
dprA upP1	ACAAGGGGTGGCTATGGCGGCAAGTC	RA ATCC11845
dprA upP2	TAAGACTGGAAAGTGGTAACTAGCGCCTTGCCAT	RA ATCC11845
ErmP1	GCAAGGCGCTAGTTACCACTTTCCAGTCTTACG	RA ATCC11845
ErmP2	GTAATTTTTCAACGACTTTGAACTACGAAGGATGAAATTTTT	RA ATCC11845
dprA downP1	TCCTTCGTAGTTCAAAGTCGTTGAAAAATTACTTTTTTAAAA	RA ATCC11845
dprA downP2	TGCTTGGCAGAATCTCATAATTTCCATATCCGA	RA ATCC11845
rpsLP1	ATGCCTACTATTCAACAATTAG	RA ATCC11845
rpsLP2	TTACTTTTTAGCATCTTTAGGACGC	RA ATCC11845
dprA CompP1	CATGCCATGGCAATGGTAAATGCGGAAGAAATT	RA ATCC11845
dprA CompP2	CCGCTCGAGCTAAATGATAGAATATCTCCTCCCA	RA ATCC11845
dprA-RFP1	CATGCCATGGCAATGATTAAAAACGAAATAAAAAT	RA ATCC11845
dprA-RFP2	CCGCTCGAGCTAAAAAAGCTCCAAAACTTTAGA	RA ATCC11845
Sp-dprAP1	CATGCCATGGCAATGGAGTTATTTATGAAAATCACAA	*S. pneumoniae* D39
Sp-dprAP2	CCGCTCGAGTTAAAATTCAAATTCCGCAAG	*S. pneumoniae* D39
Hp-dprAP1	CATGCCATGGCAGTGAATCAACGAATGAAAAGCC	*H. pylori* 26695
Hp-dprAP2	CCGCTCGAGTCACGCTAACACCACAATGTGA	*H. pylori* 26695
Fn-dprAP1	CATGCCATGGCAATGGAGCTGACGAATCCACTTGG	synthesized
Fn-dprAP2	GCTCTAGAGCTTAAGGATGAAAGCGGGCACAG	synthesized
dprA^∗^P1	AGTATTGTTGGGACGGAAAATGCCACTGCTTAT	RA ATCC11845
dprA^∗^P2	AGCAGTGGCATTTTCCGTCCCAACAATACTAAT	RA ATCC11845
dprAExP1	GGGAATTCCATATGGTAAATGCGGAAGAAATT	RA ATCC11845
dprAExP2his	CCGCTCGAGCTAGTGGTGGTGGTGGTGGTGAATGATAGAATATCTCCTCCCA	RA ATCC11845
RA-ssDNA_1_	TAGGCTCTGCTAAGGAAGCGTGGGGTCTGTCTAAGTTGGA	RA ATCC11845
RA-ssDNA_2_	AAAAACTACGGAACTGACTAAAGGCAGAAAAACTAAACGG	RA ATCC11845
EC-ssDNA	CTCAGGTGCGAAAGCGTGGGGAGCAAACAGGATTAGATAC	*E. coli* XL1-blue
dprA qRTP1	TCCGATGTTTGAGGCAATTTG	RA ATCC11845
dprA qRTP2	TGCAAGTTTGGTTAGCGAGGTAG	RA ATCC11845
recA qRTP1	CTTAGGATAACCGCCTACTC	RA ATCC11845
recA qRTP2	CTTAGGATAACCGCCTACTC	RA ATCC11845

### Media and Growth Conditions

*Riemerella anatipestifer* was routinely cultured in GC broth (GCB) with agitation (Liu et al., 2017) or on LB plates supplemented with 5% sheep blood at 37°C (Liu et al., 2018a). GCB agar plates were prepared by using GCB supplemented with 1.5% agar. *Escherichia coli* strains were grown on LB agar at 37°C. When required, antibiotics were added at the following final concentrations (μg/ml): erythromycin (Erm), 1; cefoxitin (Cfx), 1; and streptomycin (Str), 100 for *R. anatipestifer* and ampicillin (Amp), 100 for *E. coli.*

### Natural Transformation

Natural transformation was performed as described previously with slight modification (Liu et al., 2017). Briefly, the single colony was isolated on LB agar supplemented with 5% sheep blood at 37°C for 24 h. The eugonic cells were collected from the plate into GCB medium (Liu et al., 2017) and checked for OD600. Then, the bacteria were adjusted to an OD600 of 1. The bacterial suspensions (0.3 ml) were transferred to sterilized tubes, and 1 μg of plasmid DNA or genomic DNA was added to the tube. After an additional incubation for 1 h at 37°C, the bacterial cultures were serially diluted and plated onto GCB agar plates or LB agar supplemented with 5% sheep blood with or without antibiotics. Transformation frequencies were calculated as the CFU ml^−1^ on selective plates divided by the CFU ml^−1^ on non-selective plates (Kristensen et al., 2012).

### Construction of the RA ATCC11845 *dprA* Mutant

The RA ATCC11845 *dprA* mutant was constructed using the natural transformation method as described previously (Liu et al., 2017). Briefly, the upstream sequence (∼620 bp) and the downstream sequence (∼620 bp) of the *dprA* gene were amplified using the primers dprA upP1 and dprA upP2 or dprA downP1 and dprA downP2, respectively (Table 2). The 994-bp ErmR cassette was amplified from the RA-CH-1 strain (Luo et al., 2015) (Liao plos one) using the primers ErmP1 and ErmP2 (Table 2). The resulting PCR fragments were ligated using the overlapped PCR method (Xiong et al., 2006). The fused PCR fragments were purified by TianGEN (TIANGEN, Beijing, China) and introduced into the wild-type strain by natural transformation. The transformants were selected on blood agar plates supplemented with Erm (1 μg/ml). The gene-deletion mutant strain was identified by PCR by amplifying the conserved 16S rRNA gene of *R. anatipestifer* using the primers 16S rRNAP1 and 16S rRNAP2 to verify whether the mutant is *R. anatipestifer*, and the *dprA* gene was amplified using the primers dprA Comp P1 and dprA Comp P2.

### Preparation of Transformation DNA (tDNA)

Streptomycin-resistant RA ATCC11845 cells were obtained by plating 10^8^ wild-type cells on LB agar supplemented with 5% sheep blood containing 100 μg/ml streptomycin. Streptomycin-resistant clones were streaked for isolation on fresh medium, and the *rpsL* of each clone was PCR-amplified using the primers rpslP1 and rpslP2 (Table 2) and then sent for sequencing. Point mutations in *rpsL* conferring streptomycin resistance were identified by comparison to the wild type *rpsL*. Genomic DNA containing the *rpsL* mutant was extracted using the TIANamp Bacterial DNA Kit (TIANGEN, Beijing, China) and was used as the transformed DNA (tDNA). The concentration of the genomic DNA was measured by a Nanodrop2000.

### Construction of the Recombinant Vectors for Complementation

The insert fragment was cloned into the shuttle plasmid pLMF03, which contain high expression promoter, for complementation as described in previous study (Liu et al., 2016). Briefly, the entire coding region of *dprA* was amplified from RA ATCC11845 chromosomal DNA using the primers dprA Comp P1 and dprA Comp P2 (Table 2). The domain of *dprA*-SAM-RF and *dprA*-RF-Zα for *dprA* was amplified from RA ATCC11845 chromosomal DNA using the primers dprA Comp P1 and dprA-RF P2, dprA-RF P1 and dprA Comp P2, respectively (Table 2). The entire coding region of Sp-*dprA* and Hp-*dprA* was amplified from the *S. pneumoniae* D39 strain and the *Helicobacter pylori* 26695 strain using the primers Sp-dprAP1 and Sp-dprAP2, Hp-dprAP1 and Hp-dprAP2, respectively (Table 2). The entire coding region of Fn-*dprA* of *Fusobacterium naviforme* was synthesized according to the sequence (NCBI: *EI53_RS03865*) and amplified using the primers Fn-dprAP1 and Fn-dprAP2 (Table 2). The PCR products were purified, digested by the corresponding restriction endonuclease, and ligated into the plasmid pLMF03 to generate the plasmid pLMF03::*dprA*, pLMF03::*dprA*-SAM-RF, pLMF03::*dprA*-RF-Zα, pLMF03::Sp-*dprA*, pLMF03::Hp-*dprA*, and pLMF03::Fn-*dprA*.

### Construction of a Site-Directed Mutant Recombination Vector

Site-directed mutagenesis was carried out by overlap PCR to substitute the arginine residue (R) at position 123 of the *R. anatipestifer dprA* with glutamic acid (E). The desired mutant DNA fragment was obtained by two rounds of PCR. Briefly, the first 389 bp of the RA ATCC11845 *dprA* gene was amplified from genomic DNA using the primer dprA Comp P1, which contains a Nco I restriction enzyme site, and the primer dprA^∗^ P2, which contains mutant nucleotides (TTC). The second 758-bp fragment of the *dprA* gene was amplified from genomic DNA using the primer dprA^∗^ P1, which contains mutant nucleotides (GAA), and the primer dprA Comp P2, which contains an XhoI restriction enzyme site. The two fragments were then ligated by overlap PCR (Xiong et al., 2006). The resulting amplicon was digested by Nco I/XhoI and cloned into pLMF03 (also digested with NcoI and XhoI) to generate the pLMF03::*dprA*^R123E^ plasmid. The ligation products were introduced into *E. coli* strain DH5α cells using the calcium chloride method, and transformants were selected on LB plates containing Amp (100 μg/ml final concentration). The presence of the correct inserts was confirmed by PCR and sequencing (BGI, Guangzhou, China).

### Construction of RA ATCC11845Δ*dprA*::*Erm* Containing pLMF03::*dprA*, pLMF03::*dprA*-SAM-RF, pLMF03::*dprA*-RF-Zα, pLMF03::Sp-*dprA*, pLMF03::Hp-*dprA*, pLMF03::Fn-*dprA*, or pLMF03::*dprA*^R123E^ Plasmids

Because we failed to introduce the plasmid pLMF03 into RA ATCC11845Δ*dprA*::*Erm* using either natural transformation or conjugation, the plasmid was introduced into RA ATCC11845 before *dprA* was knocked out. Briefly, the pLMF03::*dprA*, pLMF03::*dprA*-SAM-RF, pLMF03::*dprA*-RF-Zα, pLMF03::Sp-*dprA*, pLMF03::Hp-*dprA*, pLMF03::Fn-*dprA*, and pLMF03::*dprA*^R123E^ plasmids was introduced into RA ATCC11845, respectively, as described previously (Huang et al., 2017). The transconjugants were selected on blood plates supplemented with Cfx (1 μg/ml). The RA ATCC11845 (pLMF03::*dprA*), RA ATCC11845 (pLMF03::*dprA*-SAM-RF), RA ATCC11845 (pLMF03::*dprA*-RF-Zα), RA ATCC11845 (pLMF03::Sp-*dprA*), RA ATCC11845 (pLMF03::Hp-*dprA*), RA ATCC11845 (pLMF03::Fn-*dprA*), and RA ATCC11845 (pLMF03::*dprA*^R123E^) strains were identified by PCR analysis. Subsequently, the *dprA* gene was deleted as described above (Liu et al., 2017). The transformants were selected on blood plates supplemented with Cfx (1 μg/ml) and Erm (1 μg/ml). The strains, which were designed RA ATCC11845Δ*dprA*::*Erm* (pLMF03::*dprA*), RA ATCC11845Δ*dprA*::*Erm* (pLMF03::*dprA*-SAM-RF), RA ATCC11845Δ*dprA*::*Erm* (pLMF03::*dprA*-RF-Zα), RA ATCC11845Δ*dprA*::*Erm* (pLMF03::Sp-*dprA*), RA ATCC11 845Δ*dprA*::*Erm* (pLMF03::Hp-*dprA*), RA ATCC11845Δ*dprA*:: *Erm* (pLMF03::Fn-*dprA*), and RA ATCC11845Δ*dprA*::*Erm* (pLM F03::*dprA*^R123E^), respectively, were identified by PCR analysis.

### Construction of the Recombinant Vector for Expression

The complete *dprA* and *dprA*^R123E^ genes were amplified by PCR from RA ATCC11845 chromosomal DNA and the recombination vector pLMF03::*dprA*^R123E^ using the primers dprAExP1 and dprAExP2his (Table 2). The resulting PCR product was purified, digested with NdeI and XhoI, and ligated into the pET30a plasmid (also digested with NdeI and XhoI) to generate pET30a::*dprA*-s and pET30a::*dprA*^R123E^-s plasmids. The ligation products were introduced into *E. coli* strain DH5α cells using the calcium chloride method, and transformants were selected on LB plates containing Kan (50 μg/ml final concentration). The presence of the correct inserts was confirmed by PCR and sequencing (BGI, Guangzhou, China).

### Expression and Purification of DprA and Dpr^R123E^ His-Tagged Proteins

The *E. coli* Rosetta (pET30a::*dprA*-s) and *E. coli* Rosetta (pET30a::*dprA*^R123E^-s) strains were grown overnight in LB medium containing Kan (50 μg/ml). Stationary-phase cultures were diluted to an OD600 of 0.05 in 500 ml of LB medium containing Kan (50 μg/ml) and then incubated with shaking at 37°C until the culture density reached an OD600 of 0.6. The cells were then induced with 0.05 mM isopropyl b-D-1-thiogalactopyranoside (IPTG) and reincubated at 25°C. The cells were harvested by centrifugation for 10 min at 8,000 rpm at 4°C, and the resulting pellet was resuspended in 50 ml of binding buffer I (50 mM Tris–HCl, 250 mM NaCl, 0.05% Triton, pH 8.0) containing lysozyme (1 mg/ml final concentration) and DNase I (1 U/ml final concentration). The bacteria were lysed by freezing and thawing the cells at least three times. The suspension was then centrifuged at 12,000 rpm for 30 min at 4°C, and the supernatant was collected. The supernatant was mixed with 250 μl of Ni-NTA-agarose beads according to the manufacturer’s instructions. Purified protein was dialyzed twice against a buffer containing 50 mM Tris–HCl to eliminate any residual imidazole. The protein was stable for at least 1 month when kept at −80°C in 20% glycerol. Protein concentrations were determined using the BCA method with bovine serum albumin as the standard.

### Electrophoretic Mobility Shift Assays (EMSA)

The DNA-binding activity of DprA and DprA^R123E^ was measured using an EMSA Kit (Beyotime, China). RA-ssDNA_1_ from RA ATCC11845 (coding sequence: TAGGCTCTGCTAAGGAAGCGTGGGGTCTGTCTAAGTTGGA), RA-ssDNA_2_ from RA ATCC11845 (non-coding sequence: AAAAACTACGGAACTGACTAAAGGCAGAAAAACTAAA CGG), *E. coli* (EC-ssDNA from *E. coli* (CTCAGGTGCGAAAGCGTGGGGAGCAAA CAGGATTAGATAC) and dsDNA annealed from RA-ssDNA_1_ were labeled with biotin using an EMSA Probe Biotin Labeling Kit (Beyotime, China). A 10 μl reaction mixture containing 0.5 μl of DNA substrate (biotin-labeled ssDNA or dsDNA) in binding buffer with 25 mM HEPES (pH 7.0), 150 mM NaCl, 10% glycerol, 1 mM DTT and 0.05% IGEPAL (v/v; Sigma-Aldrich) mixed with the indicated concentrations of DprA and DprA^R123E^. After 30 min of incubation at 30°C, the samples were electrophoresed on an 8% non-denaturing PAGE in 0.5 × TBE (45 mM Tris-borate, 1 mM EDTA, pH 8.3). A constant voltage of 10 V/cm was applied for 2 h in baths of ice. The gel was transferred to a nylon membrane (Beyotime, China) at 380 mA for 30 min in 0.5 × TBE buffer. Fluorescence detection was performed with streptavidin-conjugated HRP and BeyoECL using a Chemiluminescent EMSA Kit (Beyotime, China) as described in the product specifications.

### qRT-PCR

*Riemerella anatipestifer* ATCC11845 (pLMF03), RA ATCC11845Δ*dprA*::*Erm* (pLMF03::*dprA*), and RA ATCC11845Δ*dprA*::*Erm* (pLMF03::*dprA*^R123E^) were grown in GCB medium at an initial OD600 = 0.05 at 37°C with shaking at 180 rpm. The cells were harvested after 6 h of incubation. Total RNA was extracted using an RNAprep pure Cell/Bacteria Kit (TIANGEN, China). cDNA was synthesized from each RNA sample as described in a previous study (Liu et al., 2016). Real-time PCR assays were conducted with the primers shown in Table 2. Quantitative PCR was performed in triplicate on deposited samples as described in a previous study (Liu et al., 2016). The RNA quantity was normalized using a probe specific for 16S rRNA. Fold change was calculated as described in a previous study with the delta delta Ct method considering the efficiency of the PCR reaction for each target (Pfaffl, 2001).

### Statistics and Software

The results of the transformation experiments were analyzed using GraphPad Prism 7.0 software for Windows (GraphPad Software Inc., La Jolla, CA, United States). The homology analysis of the RF domain of DprA was based on amino acid identity and performed using ClustalX 2.0 or DNAMAN 8.0 (Lynnon Biosoft, ON, Canada). One-way ANOVA followed by Tukey’s multiple-comparison test were used to evaluate statistical significance. A *P*-value <0.05 was considered significant.

## Results

### Identification and Sequence Analysis of DprA in *R. anatipestifer* ATCC11845

**FIGURE 1:**
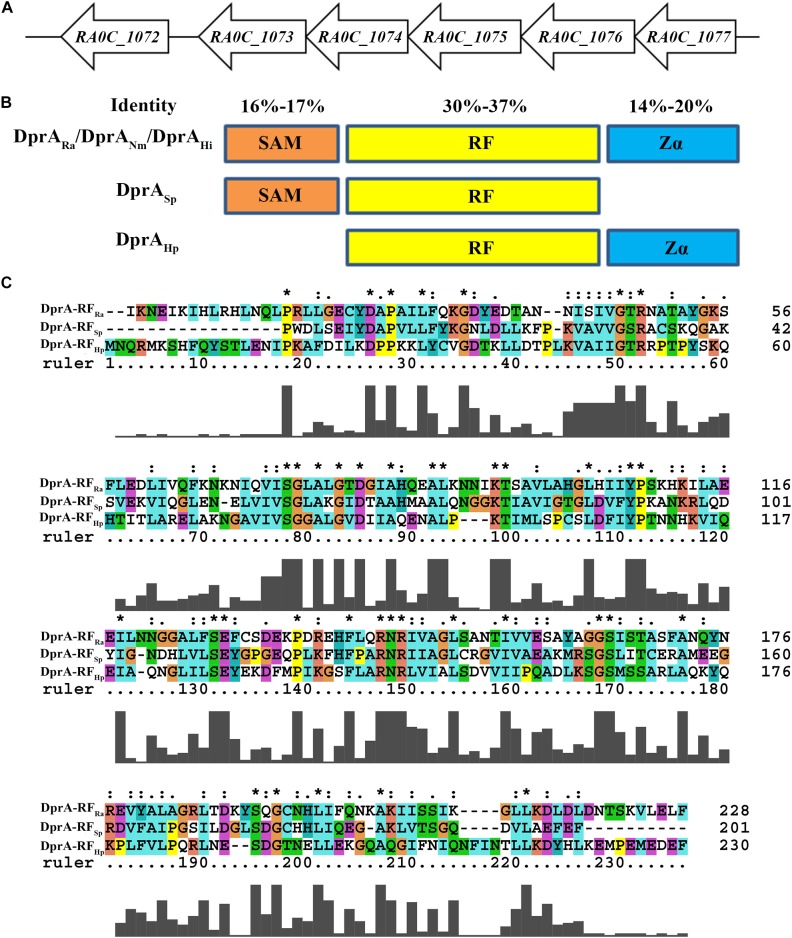
Bioinformatics analysis of DprA_Ra_. **(A)** The *dprA* locus in *R. anatipestifer* ATCC11845. ORFs are indicated by block arrows and point in the direction of transcription. Names of ORFs are indicated below each arrow. **(B)** Predicted overall domain structure of DprARa and orthologs of *S. pneumonia* (DprA_Sp_), *H. pylori* (DprA_Hp_), and *Neisseria meningitidis* (DprA_Nm_). **(C)** Deduced amino acid sequence alignment of the hallmark RF DprA domains of *R. anatipestifer* (RA), *S. pneumoniae* (Sp), and *H. pylori* (Hp); alignment was performed using ClustalX 2.0 ^∗^the same amino acid.

In the genome of RA ATCC11845, *RA0C_1073* was annotated as a putative DprA. A protein sequence comparison showed that the DprA of *R. anatipestifer* (DprA_Ra_) had low identity with other well-characterized DprA sequences, including 30% identity with the DprA of *Neisseria meningitidis* (DprA_Nm_), 31% identity with the DprA of *H. pylori* (DprA_Hp_), 37% identity with the DprA of *Streptococcus pneumoniae* (DprA_Sp_) and 36% identity with the DprA of *Haemophilus influenzae* (DprA_Hi_) The *dprA* gene (*RA0C_1073*) is the last gene in a putative operon containing *RA0C_1074*, *RA0C_1075*, *RA0C_1076*, and *RA0C_1077* in RA ATCC11845 (Figure 1A), which encode group III truncated hemoglobin, the crossover junction endodeoxyribonuclease RuvC, a hypothetical protein and an Lrp/AsnC family transcriptional regulator, respectively.

Similar to the DprA_Nm_ (Hovland et al., 2017) and DprA_Hi_ (Karudapuram et al., 1995; Karudapuram and Barcak, 1997), DprA_Ra_ has three domains: a sterile alpha motif (SAM), a Rossmann fold (RF) and a winged-helix DNA-binding motif/Z-DNA-binding domain (Zα) (Figure 1B). In contrast, DprA_Sp_ has only SAM and RF domains and lacks a Zα domain, and DprA_Hp_ has only RF and Zα domains and lacks a SAM domain (Figure 1B). Moreover, among the SAM domains of DprA_Ra_, DprA_Nm_, DprA_Sp_, and DprA_Hi_, the identity ranges from 16 to 17%; while among the Zα domains of DprA_Ra_, DprA_Nm_, DprA_Hp_, and DprA_Hi_, the identity ranges from 14 to 20%. However, for the RF domain of DprA_Ra_, DprA_Nm_, DprA_Sp_, DprA_Hp_, and DprA_Hi_, the identity ranges from 31 to 37% (Figure 1C).

### DprA Is Essential for the Natural Transformation of *R. anatipestifer* ATCC11845

In *H. influenzae*, *dprA* is necessary for the uptake of chromosomal but not plasmid DNA (Karudapuram et al., 1995). In contrast, deletion of *dprA* in *H. pylori* had an impact on transformation of both chromosomal DNA and plasmid DNA (Ando et al., 1999). To test the role of *dprA* in the natural transformation of *R. anatipestifer* ATCC11845, the mutant strain RA ATCC11845Δ*dprA*::*Erm* and a complementation strain were constructed. Transforming genomic DNA containing resistance to streptomycin as a substrate was prepared. The result showed that the transformation frequency of the wild type was 6 (±0.7) × 10^−5^. However, no transformation was detected (limit-of-detection = 4.2 (±1.5) × 10^−10^) in the *dprA* mutant, and the transformation frequency of the complemented strain was 6.3 (±1.9) × 10^−5^ (Table 3). These results suggested that the *dprA* gene of RA ATCC11845 is essential for natural transformation when genomic DNA was used as the donor. To examine whether the *dprA* of RA ATCC11845 was also involved in the uptake of plasmids, the plasmid pLMF03, which carries a cefoxitin resistance (Cfx^R^) marker (Liu et al., 2016), was used as a donor DNA to perform natural transformation. As shown in Table 3, the transformation frequency of the wild-type *R. anatipestifer* ATCC11845 strain to Cfx^R^ was 1.7 (±0.2) × 10^−7^. Again, no transformation was detected (limit-of-detection = 4.2 (±1.5) × 10^−10^) in the *dprA* mutant. These results suggest that the *dprA* of RA ATCC11845 is essential for the transformation of both genomic DNA and plasmid DNA.

**Table 3:** Natural transformation assays performed in RA ATCC11845, RA ATCC11845Δ*dprA*::*Erm*, RA ATCC11845Δ*dprA*::*Erm* (pLMF03::*dprA*), and RA ATCC11845Δ*dprA*::*Erm* (pLMF03::*dprA*^R123E^).

Strain	Transformation frequency using chromosomal DNA	Transformation frequency using plasmid DNA
RA ATCC11845	6 (±0.7) × 10^−5^	1.7 (±0.2) × 10^−7^
RA ATCC11845Δ*dprA*::*Erm*	<d.l.	<d.l.
RA ATCC11845Δ*dprA*::*Erm* (pLMF03::*dprA*)	6.3 (±1.9) × 10^−5^	NA
RA ATCC11845Δ*dprA*::*Erm* (pLMF03::*dprA*^R123E^)	< d.l.	NA

*NA, Not applicable. <d.l., below detection limit. The average detection limit of non-transformable strains was 4.2 (±1.5) × 10^−1^.*

### The Importance of Three Domains of DprA in Natural Transformation

**FIGURE 2:**
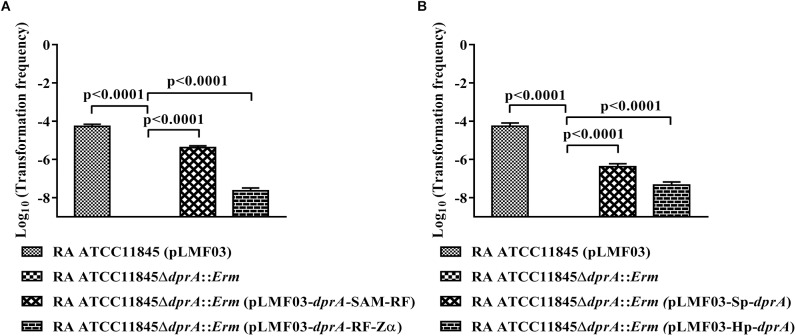
Complementation of *R. anatipestifer* ATCC11845Δ*dprA*::*Erm* by different domains of DprA_Ra_, *dprA* of *S. pneumoniae* and *dprA* of *H. pylori*. **(A)** Complementation of *R. anatipestifer* ATCC11845Δ*dprA*::*Erm* by different domains of DprA_Ra_. **(B)** Complementation of *R. anatipestifer* ATCC11845Δ*dprA*::*Erm* by *dprA* of *S. pneumoniae* (Sp-*dprA*) and *dprA* of *H. pylori* (Hp-*dprA*). The Log_10_ of averages and standard deviations of three independent experiments are shown. The numbers above each data point represent *P*-values for comparisons (one-way ANOVA followed by Tukey’s multiple-comparison test) of log_10_ of the average relative transformation frequencies.

As described above, DprA_Ra_ has three domains, similar to *N. meningitidis* and *H. influenzae*, whereas the DprA of *H. pylori* and *S. pneumoniae* have only two domains. To investigate the role of each DprA domain in natural transformation, the strain RA ATCC11845Δ*dprA*::*Erm* was complemented by a plasmid expressing SAM-RF domains or a plasmid expressing RF-Zα domains, and the natural transformation frequency was measured. As shown in Figure 2A, the transformation frequency of RA ATCC11845Δ*dprA*::*Erm* (pLMF03::*dprA*-SAM-RF) was 4.1 (±0.7) × 10^−6^, while that of RA ATCC11845Δ*dprA*::*Erm* (pLMF03::*dprA*-RF-Zα) was 2.4 (±0.5) × 10^−8^. Both partially restored the level of natural transformation in the mutant strain RA ATCC11845Δ*dprA*::*Erm*. Interestingly, the transformation frequency of RA ATCC11845Δ*dprA*::*Erm* (pLMF03::*dprA*-SAM-RF) was higher than that of RA ATCC11845Δ*dprA*::*Erm* (pLMF03::*dprA*-RF-Zα). From these results, we predicted that the role of the SAM domain is more important role than that of the Zα domain in the natural transformation of RA ATCC11845.

To further verify the functions of these three domains in natural transformation, the strain RA ATCC11845Δ*dprA*::*Erm* was complemented by either a *dprA* of *S. pneumoniae* containing SAM and RF domains or a *dprA* of *H. pylori* containing RF and Zα domains, and the natural transformation frequency was measured. As shown in Figure 2B, the transformation frequency of RA ATCC11845Δ*dprA*::*Erm* (pLMF03::Sp-*dprA*) was 3.8 (±0.9) × 10^−7^, while that of RA ATCC11845Δ*dprA*::*Erm* (pLMF03::Hp-*dprA*) was 2.9 (±0.6) × 10^−8^. Interestingly, the frequency of complementing in cells the *dprA* from *S. pneumoniae* was higher than that observed in cells with the *dprA* from *H. pylori*. These results suggest that the role of the SAM domain is more important than that of the Zα domain in natural transformation.

### The Amino Acid Arg123 in the RF Domain Is Essential for the Function of DprA in RA ATCC11845

As described above, it has been hypothesized that the RF domain is essential for the function of DprA in natural transformation. Moreover, sequence comparison showed that the amino acid residue Arg123 is one of the most conserved amino acid residues in this domain (Figure 1C). Thus, we chose to explore the function of Arg123 in natural transformation in RA ATCC11845. A mutant *dprA*^R123E^ (arginine mutated to glutamic acid) construct was developed and cloned into the shuttle plasmid pLMF03 as described in the Section “Materials and Methods”. As shown in Table 3, the plasmid pLMF03::*dprA*^R123E^ was not able to restore natural transformation to RA ATCC11845Δ*dprA*::*Erm*. As a control, *dprA*^R123E^ was transcribed well in the RA ATCC11845Δ*dprA*::*Erm* strain (pLMF03::*dprA*^R123E^; Supplementary Figure S1). The results suggested that the RF domain is essential for natural transformation in RA ATCC11845 and that the amino acid Arg123 plays a key role in this domain.

### DprA^R123E^ Has a Dramatic Effect on Binding ssDNA and dsDNA

**FIGURE 3:**
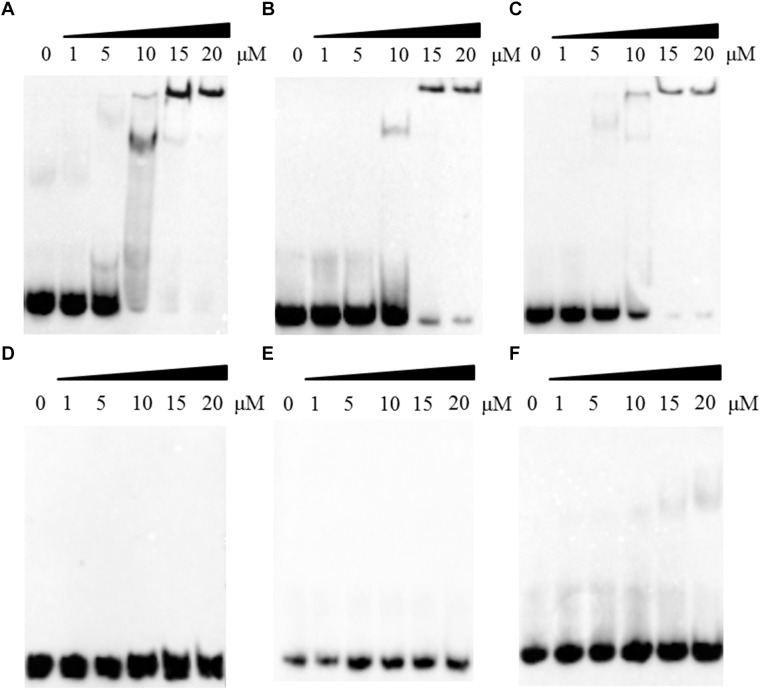
The effect of the R123E mutant of DprA on binding to single-strand DNA (ssDNA). All ssDNA was labeled by biotin. The first lane of each picture contains free ssDNA without proteins. Different concentrations of DprA (1–20 μM) were combined with 0.5 μl of DNA substrate (biotin-labeled ssDNA) and incubated for 30 min at 30°C before the mixtures were loaded onto the gel. **(A)** Interaction between DprA and ssDNA from the *R. anatipestifer* coding sequence (RA-ssDNA_1_). **(B)** Interaction between DprA and ssDNA from the *R. anatipestifer* non-coding sequence (RA-ssDNA_2_). **(C)** Interaction between DprA and ssDNA from *E. coli* XL1-blue (*E. coli*-ssDNA). **(D)** Interaction between DprA^R123E^ and ssDNA from the *R. anatipestifer* coding sequence (RA-ssDNA_1_). **(E)** Interaction between DprA^R123E^ and ssDNA from the *R. anatipestifer* non-coding sequence (RA-ssDNA_2_). **(F)** Interaction between DprA^R123E^ and ssDNA from *E. coli* XL1-blue (*E. coli*-ssDNA). Samples were electrophoresed on an 8% non-denaturing PAGE gel and detected by fluorography.

The R123E mutant of DprA abolished the ability of RA ATCC11845 to undergo natural transformation, potentially because the mutant was unable to bind and protect DNA. Thus, we next evaluated whether Arg123 of the DprA domain of RA ATCC11845 affects the ability of the protein to bind to DNA. In these experiments, DprA and DprA^R123E^ were expressed and purified, and EMSA was performed as described in the Section “Materials and Methods”. In this study, we first chose three different sources of single strand DNA (ssDNA): RA-ssDNA_1_ from the coding sequence of RA ATCC11845, RA-ssDNA_2_ from the non-coding sequence of RA ATCC11845 and (EC-ssDNA from *E. coli*. The sequences of those ssDNA were shown in the Section “Materials and Methods.” Single-strand DNA (ssDNA) was synthesized and used as a substrate for EMSA. As shown in Figure 3A–C, all the ssDNA was shifted in the presence of DprA. As the concentration of DprA increased, the formation of protein-DNA complexes was clearly slowed, and this change was associated with a parallel loss of uncomplexed DprA and ssDNA, suggesting that DprA can bind ssDNA and that it has no sequence or species specificity.

**FIGURE 4:**
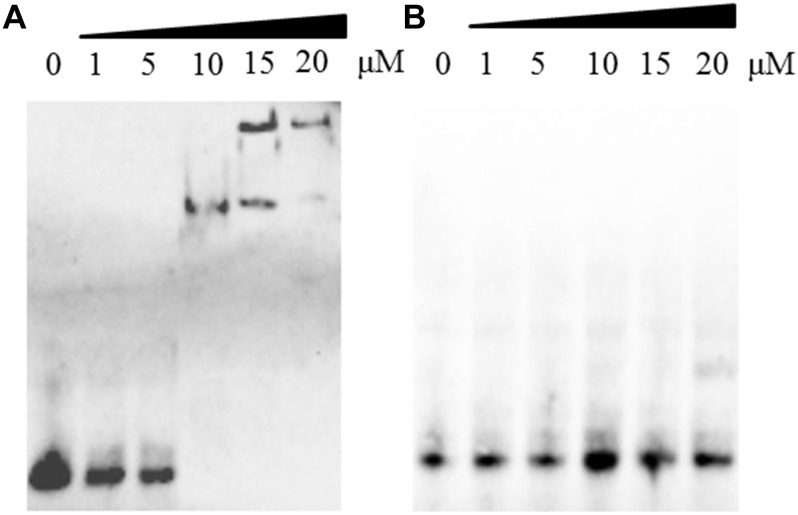
The effect of the R123E mutant of DprA on binding to double-strand DNA (dsDNA). The dsDNA was annealed from RA-ssDNA_1_ and labeled with biotin. The first lane of each picture contained free dsDNA without proteins. Different concentrations of DprA (1–20 μM) were combined with 0.5 μl of DNA substrate (biotin-labeled dsDNA) and incubated for 30 min at 30°C before the mixture was loaded on the gel. **(A)** Interaction between DprA and dsDNA. **(B)** Interaction between DprA^R123E^ and dsDNA. Samples were electrophoresed on an 8% non-denaturing PAGE gel and detected by fluorography.

Interestingly, DprA of *H. pylori* binds not only ssDNA but also dsDNA (Dwivedi et al., 2013). However, DprA of *S. pneumoniae* and *Bacillus subtilis* has been reported to bind and protect ssDNA but not dsDNA (Mortier-Barriere et al., 2007). To determine whether DprA of *R. anatipestifer* binds dsDNA, EMSA was performed using both DprA and biotinylated dsDNA. The dsDNA (40 bp) was annealed from RA-ssDNA_1_. As shown in Figure 4A, the retarded protein-DNA complex became more evident with the increasing of DprA concentration, suggesting that DprA binds dsDNA. As shown in Figure 3D–F, 4B, there is not any retarded protein-DNA complex as the concentration of the DprA^R123E^ was increased as the concentration of DprA^R123E^ increased. Taken together, these results suggest that the Arg123 mutant abolished the ability of DprA to bind and protect DNA.

### DprA Has a Conserved Function in Evolution

**FIGURE 5:**
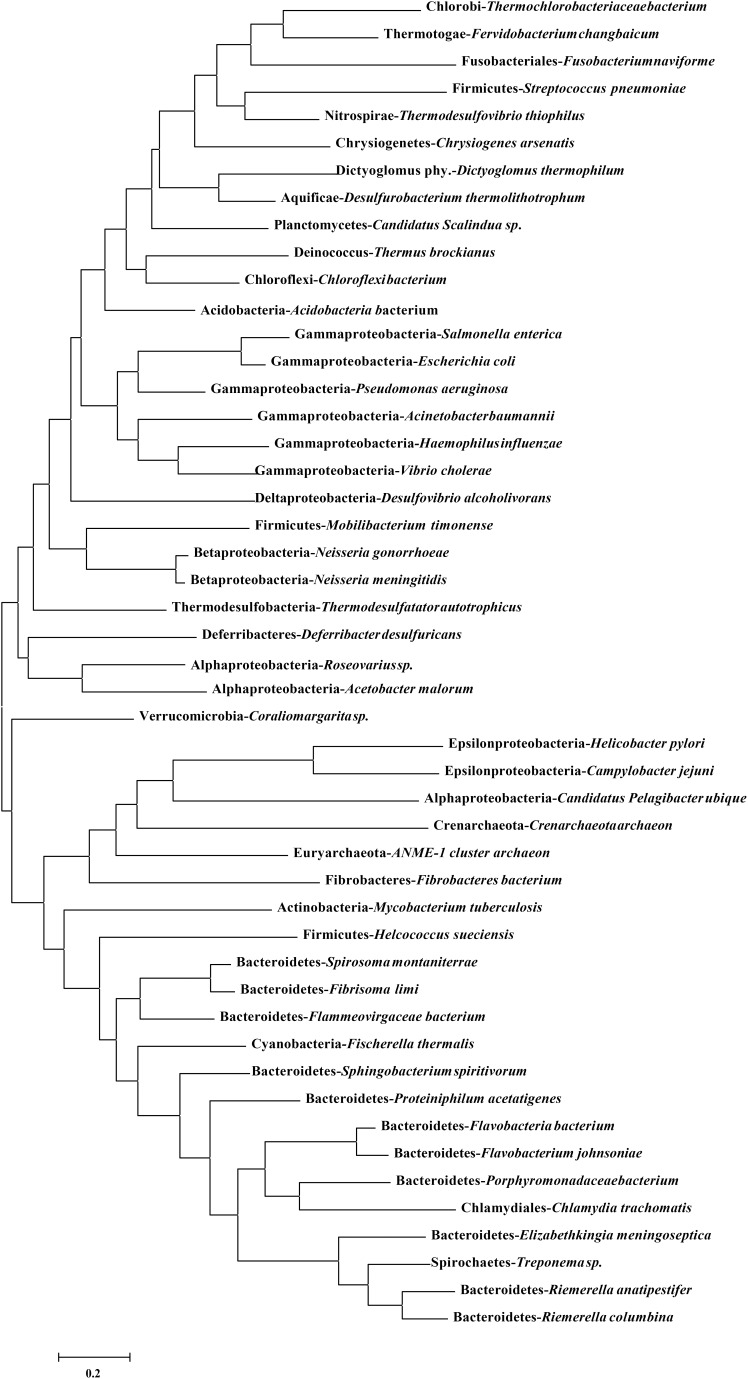
Phylogenetic analysis of DprA among different bacterial species. A phylogenetic tree was constructed based on amino acid sequences using MEGA6.0 using neighbor-joining method. The sequence information for DprA is listed in Supplementary Table S1.

**FIGURE 6:**
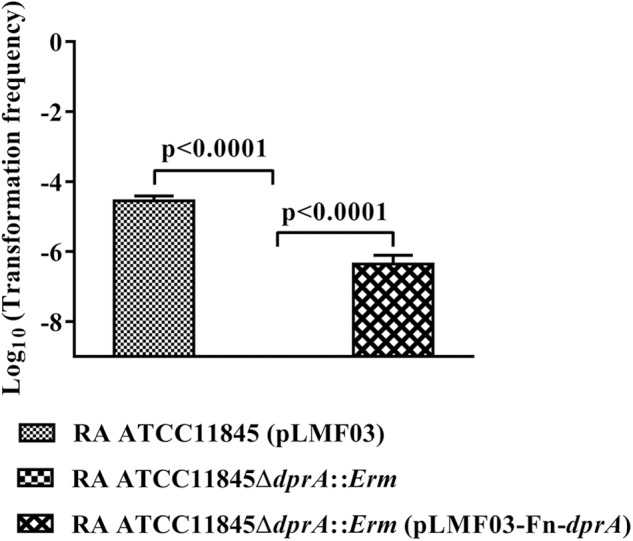
Complementation of *R. anatipestifer* ATCC11845Δ*dprA*::*Erm* by the *dprA* of the non-competent bacterium *F. naviforme*. The Log_10_ of averages and standard deviations of three independent experiments are shown. The numbers above each data point represent *P*-values for comparisons (one-way ANOVA followed by Tukey’s multiple-comparison test) of log_10_ of the average relative transformation frequencies.

DprA has been found in nearly all the sequenced bacteria, including bacteria currently recognized as non-competent (Supplementary Table S1). The identity ranged from 35 to 63% in *Flavobacteriaceae* (Supplementary Table S1). Compared to other bacterial genera, for RA ATCC11845, the identity ranged from 28 to 39%, but it shared 59% identity with *Spirochaetes*. As shown in Figure 5, a phylogenetic analysis of DprA across different bacterial species showed that there were no obvious branches between naturally competent bacteria and non-competent bacteria. It has been speculated that DprA has conserved functions across different bacteria. Thus, we wondered whether *dprA* from other bacteria would be able to restore natural transformation to RA ATCC11845Δ*dprA*::*Erm*. In a proof-of-principle experiment, we chose the DprA of *F. naviforme*, which has not been reported natural competence currently and has low identity (30%) with the DprA of RA ATCC11845 (Supplementary Figure S3). The *dprA* of *F. naviforme* was synthesized and cloned into the shuttle plasmid pLMF03. An RA ATCC11845Δ*dprA*::*Erm* construct that expressed pLMF03::Fn-*dprA* was constructed as described in the Section “Materials and Methods”. As shown in Figure 6, the transformation frequency of RA ATCC11845 (pLMF03) was 2.9 (±0.3) × 10^−5^, while that of RA ATCC11845Δ*dprA*::*Erm* (pLMF03::Fn-*dprA*) was 4.9 (±0.7) × 10^−7^. These results suggest that a *dprA* from *F. naviforme*, partially restored natural transformation to the mutant RA ATCC11845Δ*dprA*::*Erm*.

## Discussion

Natural transformation is a widely distributed mechanism used by many bacterial genera to acquire DNA and undergo genetic recombination (Johnston et al., 2014). The competence machinery actively processes exogenous dsDNA and takes up internalized ssDNA to replace homologous (or partially homologous) chromosomal sequences via a mechanism catalyzed by RecA and facilitated by accessory factors, such as DprA (Kidane et al., 2012). Although it has been found that *R. anatipestifer* was natural competent and established multiple genome editing tools using it (Liu et al., 2018b), the mechanism of natural transformation in *R. anatipestifer* kept largely unknown. This study focused on the characterization of DprA in *R. anatipestifer* ATCC11845.

DprA is essential for the natural transformation in *N. meningitidis*, *N. gonorrhoeae*, *S. pneumoniae*, and *H. influenzae* (Karudapuram et al., 1995; Berge et al., 2003; Duffin and Barber, 2016; Hovland et al., 2017). However, in *B. subtilis*, *dprA* is not stringently required for DNA transformation as there is some redundancy between the RecF and DprA pathways (Kidane et al., 2009). *RA0C_1073* of RA ATCC11845 encodes a hypothetical DprA with low identity to the DprA sequences of other bacteria. To explore whether this gene is involved in natural transformation in RA ATCC11845, a *dprA* mutant strain was constructed. The transformation frequency of *dprA* mutant strain was not able to be detected whatever plasmid or chromosomal DNA was used as the donor DNA, suggesting that DprA is essential for the natural transformation of both chromosomal DNA and plasmid DNA in RA ATCC11845, consistent with what has been observed in *H. pylori* (Ando et al., 1999), but inconsistent with *H. influenzae*, in which *dprA* is necessary for chromosomal uptake but not for plasmid (Karudapuram et al., 1995). In the case of *H. influenzae*, it was hypothesized that the plasmid DNA enters the cytoplasm without cutting and recombination. However, in the case of *R. anatipestifer*, we predicted that the plasmid DNA enter the cytoplasm as the single strand DNA and was re-circled, since if the circular DNA enters into the cytoplasm directly, the *dprA* mutant should have no any effect on transformation frequency of plasmid. However, the fact is that the plasmid was not able to be introduced into the *dprA* mutant by natural transformation after many times attempts. Additionally, we tried many times to introduce the plasmid into *dprA* mutant by conjugation. It was also failed. Although it is impossible that natural transformation and conjugation use the same pathway, it is predicted that single-stranded linear of the plasmid is transported into the cytoplasm by conjugation and the *dprA* was required for the recyclizing.

Bioinformatic analysis showed that DprA_Ra_ has three domains (SAM, RF and Zα domains). To investigate the function of each domain of DprA, complementary assays were performed with SAM-RF domain and RF-Zα domain, respectively. It was showed that the natural transformation frequency was higher when the mutant strain was complemented by SAM-RF domain, than that complemented by RF-Zα domain. Consistently, the *dprA* of *H. pylori* (Hp-*dprA*), which contains SAM and RF domains, was more efficiently to restore the natural transformation of the *dprA* mutant strain than *dprA* of *S. pneumoniae* (Sp-*dprA*), which contains RF and Zα domain, did. All these results suggest that the SAM domain is more important than Zα domain for natural transformation. It is possible that the SAM domain play more important role than Zα domain in protect ssDNA or promote recombination. In DprA_Sp_, the SAM domain plays a role in intracellular signaling and the regulation of competence, in which it plays an important role in shutting down natural competence in this bacterium by interacting with ComE (Mirouze et al., 2013). Compared with *S. pneumonia*, there are some different traits in *R. anatipestifer*. First, the natural competence of *R. anatipestifer* is constitutive. Second, the SAM domain in DprA_Ra_ lacks the amino acid residues that confer the ability to induce competence in *S. pneumoniae* (Supplementary Figure S2). Third, *R. anatipestifer* lacks the ComDE two-component system, which is responsible for regulating natural competence in *S. pneumoniae* (Martin et al., 2013). The specific function that the SAM domain of RA ATCC11845 plays in natural transformation is unknown at this time. It has been hypothesized that the SAM domain of DprA in *R. anatipestifer* protects ssDNA from degradation or interacts with RecA to promote homologous recombination.

When the conserved amino acid Arg123 in the RF domain of DprA of RA ATCC11845 was switched to glutamic acid (Glu), the resulting Arg123 mutant completely abolished the ability of RA ATCC11845 to undergo natural transformation, suggesting that RF is essential for natural transformation in *R. anatipestifer*. It has also been shown that Arg123 is essential for the function of DprA_Ra_. To explore whether the abrogation of natural transformation observed in the Arg123 mutant strains was because the mutant was no longer able to bind DNA, EMSA was performed to evaluate the interaction between ssDNA and DprA or DprA^R123E^. The results showed that DprA binds ssDNA without sequence or species specificity. Moreover, DprA can also bind dsDNA, consistent with *H. pylori* (Dwivedi et al., 2013). As expected, DprA^R123E^ was no longer able to bind either ssDNA or dsDNA. In this study, it was shown that RF was essential for natural transformation and that the Arg123 site in the RF domain was essential for the functions of DprA in *R. anatipestifer* ATCC11845, which is consistent with findings in *H. pylori*. In *H. pylori*, the 3D structure of DprA was studied, and the results showed that Arg52 is essential for DprA_Hp_ to grasp the substrate with high affinity (Wang W. et al., 2014).

Phylogenetic assay showed that the homolog of DprA was distributed in most of sequenced bacterial species, including the bacteria that was not found to be natural competent at present. It was consistent with the previous study which suggested that natural competence is an ancient ancestral trait and is usually to be lost during the evolution (Redfield et al., 2006). This viewpoint was strengthened by the fact that the *dprA* of *F. naviforme*, which has not been reported natural competence currently, was able to partially restored natural transformation to *R. anatipestifer dprA* mutant. It has been reported that DprA interact with RecA to promote recombination (Mortier-Barriere et al., 2007). There is a possible reason for explaining why *dprA* of *F. naviforme* only partially restore the ability of natural competence of *R. anatipestifer*. Taken together, *R. anatipestifer* is the first bacterium to be identified as a naturally competent species in *Flavobacteriaceae*. In this study, our experiments involving DprA were helpful for revealing the mechanism of natural transformation in *R. anatipestifer*, even in *Flavobacteriaceae*. These findings will also be helpful for improving gene editing methods for *R. anatipestifer*.

## Author Contributions

ML, AC, and FB conceived and designed the experiments. LH, XT, DZ, MW, YL, LZ, XC, and YY performed the experiments. LP, MR, JH, RJ, SC, and XZ analyzed the data. BT, YW, QY, and SZ contributed reagents, materials, and analysis tools. LH, ML, FB, and AC wrote the manuscript. All authors have reviewed the manuscript.

## Conflict of Interest Statement

The authors declare that the research was conducted in the absence of any commercial or financial relationships that could be construed as a potential conflict of interest.
