# Supplementary material for: DprA Is Essential for Natural Competence in Riemerella anatipestifer and Has a Conserved Evolutionary Mechanism
Source: Front Genet. 2019 May 17;10:429. doi: 10.3389/fgene.2019.00429 (PMC6533540; doi:10.3389/fgene.2019.00429)
Supplement: Supplementary file 1 [file Data_Sheet_1.PDF]

Supplementary Material

Table S1 The distribution of DprA in bacteria

Phylum	Genus	Strains	Protein ID	Query Cover	Identity
**Bacteroidetes**	*Flavobacteriia*	*Flavobacteria bacterium* BAL38	WP_040470336	98%	40%
		Elizabethkingia meningoseptica	WP_078782936	98%	56%
		Flavobacterium johnsoniae	WP_012023081	98%	38%
	Bacteroidia	*Porphyromonadaceae bacterium*	WP_036828764	97%	41%
		Proteiniphilum acetatigenes	WP_019541030	92%	37%
	Cytophagia	*Flammeovirgaceae bacterium 311*	WP_061991895	98%	35%
		Spirosoma montaniterrae	WP_077131073	97%	35%
		Fibrisoma limi	WP_009283384	98%	38%
	Riemerella	*Riemerella anatipestifer*	WP_004918085	100%	100%
		Riemerella columbina	WP_018675493	99%	63%
	Sphingobacteriia	*Sphingobacterium spiritivorum*	WP_003007972	97%	39%
**Proteobacteria**	*Alphaproteobacteria*	*Roseovarius sp.TM1035*	WP_083803295	95%	31%
		Acetobacter malorum	WP_061505003	58%	34%
		Candidatus Pelagibacter ubique	WP_050748704	39%	36%
	Betaproteobacteria	*Neisseria gonorrhoeae*	WP_050156549	79%	31%
		Neisseria meningitidis	WP_049322233	95%	31%
	Gammaproteobacteria	*Pseudomonas aeruginosa*	WP_033997708	73%	34%
		Salmonella enterica	WP_080079733	71%	34%
		Haemophilus influenzae	WP_049375009	75%	37%
		Vibrio cholerae	WP_057558091	80%	36%
		Escherichia coli	WP_000228541	81%	35%
		Acinetobacter baumannii	WP_032876250	71%	34%
	Deltaproteobacteria	*Desulfovibrio alcoholivorans*	WP_029457608	52%	34%
	Epsilonproteobacteria	*Helicobacter pylori*	WP_002208200	62%	31%
		Campylobacter jejuni	WP_002875504	48%	36%
**Actinobacteria**	*Actinobacteria*	*Mycobacterium tuberculosis*	WP_086153916	85%	28%
**Firmicutes**	*Bacilli*	*Streptococcus pneumoniae*	WP_001814244	55%	37%
	Clostridia	*Mobilibacterium timonense*	WP_077390961	76%	32%
	Tissierellia	*Helcococcus sueciensis*	WP_027347736	87%	35%
Verrucomicrobia	*Opitutae*	*Coraliomargarita sp.*	WP_110129754	92%	33%
**Deinococcus**	*Deinococci*	*Thermus brockianus*	WP_071676598	75%	31%
**Acidobacteria**	*Acidobacteria*	*Acidobacteria bacterium*	PYU95179	98%	33%
Fusobacteria	*Fusobacteriales*	*Fusobacterium naviforme*	WP_106611841	82%	31%
Dictyoglomus	*Dictyoglomales*	*Dictyoglomus thermophilum*	WP_012547757	80%	35%
Fibrobacteres	*Fibrobacteres*	*Fibrobacteres bacterium*	OIP46473	75%	34%
Spirochaetes	*Spirochaetales*	*Treponema sp.*	WP_104793058	98%	59%
Chlamydiae	*Chlamydiales*	*Chlamydia trachomatis*	CQB87891	94%	36%
Planctomycetes	*Planctomycetia*	*Candidatus Scalindua*	WP_096896230	94%	33%
**Chlorobi**	*Chlorobia*	*Candidatus Thermochlorobacteriaceae bacterium GBChlB*	KER09968	96%	32%
**Cyanobacteria**	*Nostocales*	*Fischerella thermalis*	PMB20972	98%	37%
Deferribacteres	*Deferribacteres*	*Deferribacter desulfuricans*	WP_013008505	76%	34%
Nitrospirae	*Nitrospirales*	*Thermodesulfovibrio thiophilus*	WP_028845145	97%	34%
Chloroflexi	*Chloroflexi*	*Chloroflexi bacterium*	OGO45108	97%	35%
Chrysiogenetes	*Chrysiogenetes*	*Chrysiogenes arsenatis*	WP_084417717	71%	33%
Thermodesulfobacteria	*Thermodesulfobacteria*	*Thermodesulfatator autotrophicus*	WP_068541233	98%	33%
Thermotogae	*Thermotogae*	*Fervidobacterium changbaicum*	WP_090221777	82%	33%
Aquificae	*Aquificae*	*Desulfurobacterium thermolithotrophum*	WP_013637693	80%	35%
**Euryarchaeota**	*Methanomicrobia*	*ANME-1 cluster archaeon*	PXF52960	52%	39%
Crenarchaeota	*Crenarchaeota*	*Crenarchaeota archaeon*	OLD11621	44%	33%

Note: Phyla shown in bold represent those with natural transformation.
