# Supplementary material for: DprA Is Essential for Natural Competence in Riemerella anatipestifer and Has a Conserved Evolutionary Mechanism
Source: Front Genet. 2019 May 17;10:429. doi: 10.3389/fgene.2019.00429 (PMC6533540; doi:10.3389/fgene.2019.00429)
Supplement: Supplementary file 2 [file Data_Sheet_2.pdf]

Supplementary Material

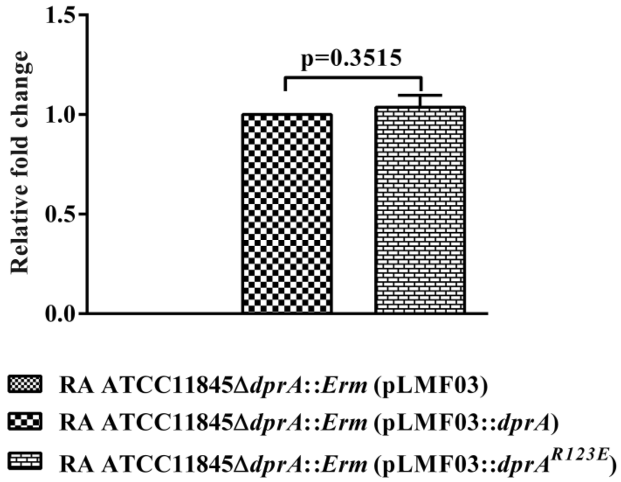

Supplementary Figure 1. The transcription levels of *dprA* and *dprA*^{R123E} in RA ATCC11845Δ*dprA*::*Erm*, RA ATCC11845Δ*dprA*::*Erm* (pLMF03::*dprA*) and RA ATCC11845Δ*dprA*::*Erm* (pLMF03::*dprA*^{R123E}). Fold-changes were calculated with the delta-delta Ct method to determine the efficiency of PCR for each target gene. The error bars represent the standard deviations of three independent experiments. The RNA quantity was normalized using a probe specific for 16s rRNA.
