# Supplementary material for: DprA Is Essential for Natural Competence in Riemerella anatipestifer and Has a Conserved Evolutionary Mechanism
Source: Front Genet. 2019 May 17;10:429. doi: 10.3389/fgene.2019.00429 (PMC6533540; doi:10.3389/fgene.2019.00429)
Supplement: Supplementary file 4 [file Data_Sheet_4.pdf]

Supplementary Material

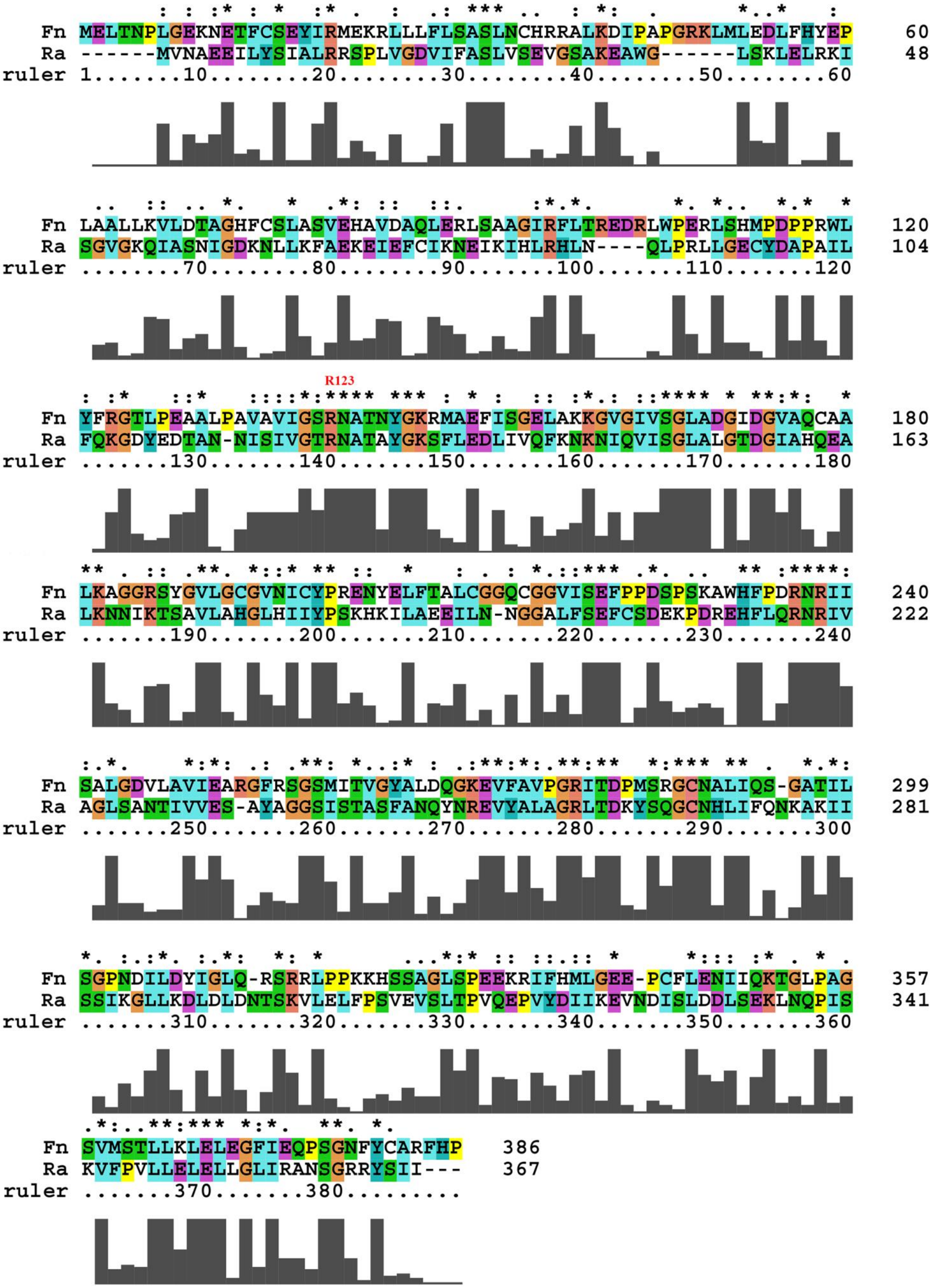

Supplementary Figure 3. Amino acid sequence alignment of DprA_{Ra} and $\text{DprA}_{\text{Fn.}}$ R123 site is conserved between these two proteins.
