# Supplementary material for: DprA Is Essential for Natural Competence in Riemerella anatipestifer and Has a Conserved Evolutionary Mechanism
Source: Front Genet. 2019 May 17;10:429. doi: 10.3389/fgene.2019.00429 (PMC6533540; doi:10.3389/fgene.2019.00429)
Supplement: Supplementary file 3 [file Data_Sheet_3.pdf]

Supplementary Material

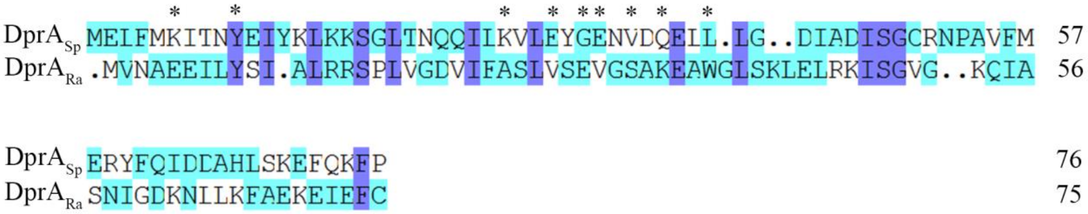

Supplementary Figure 2. Amino acid sequence alignment of the SAM domains of DprA$_{Sp}$ and DprA$_{Ra}$. * Residues involved in competence shut-off in *S. pneumonia*.
